# Not All ST Elevation Is STEMI: Brugada Phenocopy Induced by Hyperkalemia

**DOI:** 10.7759/cureus.36951

**Published:** 2023-03-31

**Authors:** Ermias S Greffie, Saleh Alhalaseh, Lynn Zaremski

**Affiliations:** 1 Internal Medicine, St. Barnabas Hospital (SBH) City University of New York (CUNY) School of Medicine, Bronx, USA; 2 Medicine/Cardiology, St. Barnabas Hospital (SBH) City University of New York (CUNY) School of Medicine, Bronx, USA

**Keywords:** st elevation, brugada ekg pattern, brugada syndrome, hyperkalemia, brugada phenocopy

## Abstract

Brugada syndrome (BrS) is a hereditary channelopathy associated with malignant ventricular arrhythmia and sudden death in individuals with a structurally normal heart. It is characterized by an ST-segment elevation in the precordial leads. Brugada phenocopy (BrP) is a term given to conditions that could result in ST morphologies identical to those found in Brugada syndrome (Brugada pattern electrocardiogram (EKG) changes) without the actual channelopathy responsible for Brugada syndrome. BrP is a rare EKG manifestation of hyperkalemia, commonly seen at high serum levels of potassium, and associated with malignant arrhythmia. Here, we present a case with Brugada pattern EKG changes associated with hyperkalemia and metabolic acidosis, which normalized after correcting the electrolyte abnormalities. In this case, we also wanted to highlight that not all ST-segment elevation is due to myocardial infarction (MI). In young patients with no coronary artery disease (CAD) risk factors, other potential ST elevation causes should be considered.

## Introduction

Brugada syndrome (BrS) is an uncommon and genetically predisposed form of cardiac arrhythmia primarily affecting younger males [1]. On electrocardiogram (EKG), it usually appears as an atypical right bundle branch block with ST elevation in the precordial leads. On the other hand, Brugada phenocopy (BrP) is characterized by an EKG pattern similar to that seen in BrS, which is induced by various clinical settings, including, but not limited to, metabolic and electrolyte abnormalities in the absence of congenital ion channel dysfunction [2]. The Brugada EKG pattern usually resolves after the underlying cause is identified. In the following case, we evaluate a patient with a Brugada-like EKG pattern in which the correction of metabolic derangements leads to the resolution of the previous EKG pattern.

## Case presentation

**Figure 1:**
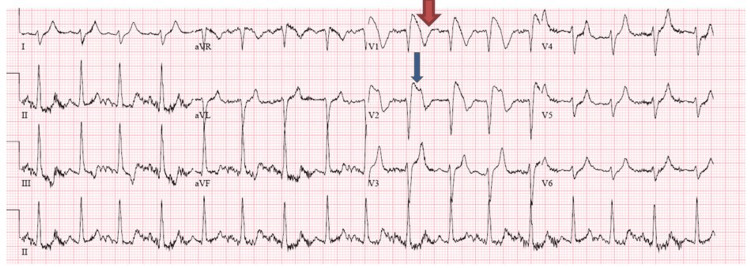
Initial EKG showing evidence of ST-segment elevation in leads V1- V2 with T wave inversion in the same leads (type 1 Brugada pattern) The wide red arrow shows T wave inversion in V1. The thin blue arrow shows ST-segment elevation in V2. EKG: electrocardiogram

A 29-year-old Hispanic male with a past medical history of polysubstance abuse was brought to the emergency department (ED) by emergency medical services (EMS) after being found unconscious in his bedroom by his father. Naloxone was administered in the field by EMS, after which he regained consciousness temporarily. However, later, he became bradycardic and suffered a cardiac arrest with an initial rhythm of asystole. Advanced cardiac life support (ACLS) was initiated, the patient was intubated in the field, epinephrine was administered three times, and the patient’s rhythm eventually became ventricular fibrillation, for which he received one shock; after that, return of spontaneous circulation (ROSC) was achieved in 15 minutes. Upon arrival in the ED, he had another two episodes of cardiac arrest, after which he was successfully resuscitated after 30 minutes but never regained consciousness. The initial vital signs in the ED were as follows: blood pressure of 130/70 mmHg, heart rate of 90 beats/minute, temperature of 97.3°F (36.2°C), and 99% O2 saturation on mechanical ventilation. Electrocardiogram (EKG) performed in the ED showed 5 mm ST elevation in V1 and V2, with T wave inversion in the same leads (Figure 1).

The differential diagnosis included acute myocardial infarction (MI) and Brugada pattern due to severe metabolic derangement and electrolyte imbalance. As shown in Table 1, the initial arterial blood gas had severe metabolic acidosis. Relevant serum chemistry tests are shown in Table 2. The test was significant for hyperkalemia, elevated blood urea nitrogen (BUN), creatinine, creatine phosphokinase (CPK), and troponin.

**Table 1:** Initial and subsequent arterial blood gas profile PCO2: partial pressure of carbon dioxide, PO2: partial pressure of oxygen, HCO3⁻: bicarbonate, FIO2: fraction of inspired oxygen

Test	Day 1	Day 2	Day 3	Day 4	Day 5	Reference range
pH	6.78	7.34	7.21	7.37	7.10	7.350-7.450 pH units
PCO2	87 mmHg	31 mmHg	50.6 mmHg	40.8 mmHg	92.2 mmHg	32-43 mmHg
PO2	74 mmHg	160 mmHg	117 mmHg	204 mmHg	394 mmHg	72-104 mmHg
Lactic acid	21 mmol/L	7.6 mmol/L	1.3 mmol/L	4 mmol/L	2 mmol/L	0.5-2.2 mmol/L
HCO3⁻	6.7 mEq/L	18 mEq/L	17.8 mEq/L	23 mEq/L	21 mEq/L	22-26 mEq/L
FIO2	21%	60%	50%	100%	100%	%

**Table 2:** Initial and subsequent relevant clinical chemistry tests BUN: blood urea nitrogen, CPK: creatine phosphokinase

Test	Day 1	Day 2	Day 3	Day 4	Day 5	Reference range
K+	8.2 mEq/L	4.8 mEq/L	8.3 mEq/L	6.5 mEq/L	3.5 mEq/L	3.5-5.3 mEq/L
Creatinine	3.7 mg/dL	4.8 mg/dL	5.6 mg/dL	4.5 mg/dL	2.4 mg/dL	0.6-1.2 mg/dL
BUN	37 mg/dL	54 mg/dL	56 mg/dL	32 mg/dL	23 mg/dL	8-23 mg/dL
Troponin	1.09 ng/mL, after six hours: 4.46 ng/mL	8.2 ng/mL, after six hours: 6.11 ng/mL	-	8.27 ng/mL	-	0-0.49 ng/dL
CPK	281,338 IU/L	286,080 IU/L	-	248,366 IU/L	153,206 IU/L	38-174 IU/L

**Figure 2:**
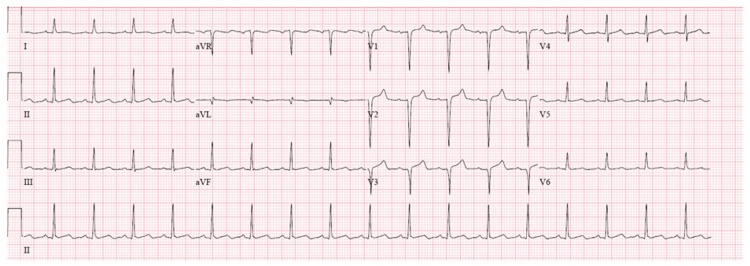
Subsequent EKG obtained after the resolution of hyperkalemia showing the resolution of the ST-segment elevations and T wave inversions EKG: electrocardiogram

Urine toxicological screening was positive for cocaine and opiates. The blood alcohol level was negative. The case was discussed with interventional cardiology, but cardiac catheterization was deferred due to multiple cardiac arrests and severe metabolic derangements. The patient received treatment for hyperkalemia with 50% dextrose, insulin, albuterol, and calcium gluconate. He also received sodium bicarbonate for metabolic acidosis. The hyperkalemia was initially corrected, and subsequent EKG showed complete resolution of the ST elevations and T wave inversion, essentially returning to a normal EKG (Figure 2).

The patient was then transferred to the intensive care unit (ICU), and targeted temperature management (TTM) for cardiac arrest was started. He was later found to have compartment syndrome of the right leg with rhabdomyolysis, likely due to lying on the affected limb for a long time while unconscious after illicit drug abuse. The creatine phosphokinase (CPK) level trended up, and the course was complicated by acute kidney injury, recurrent hyperkalemia, and acidosis, for which he received aggressive medical therapy. Fasciotomy was also performed on the right leg. Despite all these measures, controlling hyperkalemia and metabolic acidosis was challenging. The patient had two more episodes of cardiac arrest with ventricular fibrillation, for which he was defibrillated. He was finally placed on continuous renal replacement therapy (CRRT) for refractory metabolic acidosis and hyperkalemia. The subsequent EKGs were normal and did not show the Brugada pattern again or any ischemic changes. The troponin, on the other hand, was trending up. While it was possible to control the metabolic and electrolyte abnormalities with CRRT, the patient never regained consciousness. Computed tomography (CT) scan of the brain showed evidence of severe anoxic-ischemic encephalopathy with massive cerebral and cerebellar edema complicated by central herniation. All brain stem reflexes were negative. After discussing the prognosis with the next of kin, an apnea test was done to confirm brain death, and the patient was declared dead and disconnected from life support.

## Discussion

Brugada syndrome is a familial channelopathy associated with sudden cardiac death in young adults. It has characteristic EKG patterns that could be classified into two types. The most frequent pattern is the type 1 (“coved”) EKG pattern, which is characterized by a high takeoff ST-segment elevation in the right precordial leads (V1 and V2), followed by a down-sloping concave or rectilinear ST with a negative and symmetric T wave. The less common type 2 EKG pattern (“saddleback”) is characterized by an ST-segment that has a “saddleback” ST-T wave configuration, in which the elevated ST-segment descends toward the baseline and then rises again to an upright or biphasic T wave. In individuals with Brugada syndrome, the EKG pattern can spontaneously be seen on all EKGs. Sometimes, patients with Brugada syndrome may have a normal EKG only to uncover the Brugada pattern under certain circumstances such as a high fever. A Brugada syndrome diagnosis can be made in these individuals if the Brugada pattern is seen after a provocative test with intravenous sodium channel blockers such as ajmaline (ajmaline challenge test) [3].

Brugada syndrome diagnosis is suspected in individuals with characteristic EKG patterns after ruling out other potential causes. Many metabolic and cardiac abnormalities can produce the Brugada pattern EKG changes. Different terminologies have been used to describe these heterogeneous conditions, such as “acquired Brugada syndrome” and “Brugada pattern EKG.” Recently, these have collectively been named Brugada phenocopy. It is paramount to differentiate Brugada phenocopy from Brugada syndrome as the management and prognosis are different [4]. Most causes of Brugada phenocopy are reversible and do not require an implantable cardiac defibrillator (ICD). On the other hand, true Brugada syndrome requires the insertion of an ICD to prevent malignant arrhythmias and sudden cardiac death [4]. While different causes of Brugada phenocopy have been described in the literature, metabolic and electrolyte abnormalities are most commonly implicated [5]. The reported metabolic causes included hyponatremia, hypokalemia, hyperkalemia, hypophosphatemia, hypothermia, diabetic ketoacidosis, and hypopituitarism [6].

Our patient’s initial EKG showed a type 1 Brugada pattern with the characteristic ST-segment elevation and T wave inversions in leads V1 and V2, which later wholly resolved. There are two possibilities here. It is possible that the patient had a Brugada phenocopy solely due to metabolic derangements (hyperkalemia and metabolic acidosis). On the other hand, it is difficult to rule out an underlying Brugada syndrome due to a channelopathy that is unmasked by hyperkalemia. Ideally, a provocative drug test should differentiate between the two [5]. In our case, the patient was not stable enough to do the provocative drug test. Nonetheless, the fact that it wholly resolved after the normalization of potassium and the absence of a family history of sudden cardiac death as reported by the family suggests that this EKG pattern likely represented a BrP rather than a BrS. Although the Brugada pattern has been reported as one of the EKG changes in hyperkalemia, it is generally a rare finding. It is usually seen at high serum potassium concentrations level and is a life-threatening condition associated with malignant arrhythmia. An analysis of the Brugada phenocopy registry reported that out of 119 cases of BrP, 27 (22.6%) had hyperkalemia. The reported mean serum potassium level in these patients was 7.45 ± 0.89 mmol/L. The EKG pattern resolved within hours after the correction of the potassium level with a median time to resolution of seven hours. A provocative challenge test was done in seven (28%) patients and failed to reproduce the pattern excluding underlying BrS [7].

The pathophysiology of the Brugada EKG pattern is poorly understood, but it has been associated with inhibiting sodium channels with a predominant outward potassium current. These distortions of normal ion currents generate a voltage gradient between the endocardium and epicardium of the right ventricular outflow tract (RVOT), manifesting as a Brugada pattern on the EKG [6,7]. A modeling study showed that membrane depolarization and reduced sodium channel availability leading to delayed and heterogeneous conduction, particularly in the presence of fibrosis at the right ventricular outflow tract, was responsible for BrP in hyperkalemia [8].

In our patient, there was an initial suspicion of ST elevation myocardial infarction as there was an ST-segment elevation on the EKG and elevated troponin. The Brugada-type EKG pattern is one of the differential diagnoses for ST-segment elevation in anterior leads. It should also be noted that hyperkalemia can also cause ST-segment abnormalities. Troponin elevation has also been observed in patients with severe rhabdomyolysis, and there is controversy about whether it signifies cardiac ischemia [9]. Although coronary angiography was not performed, the fact that the EKG abnormalities resolved with the correction of the metabolic abnormalities and the absence of other risk factors speak against obstructive coronary artery disease (CAD). With this case, we wanted to highlight that not all ST-segment elevation is due to myocardial infarction. In young patients with no risk factors, the search should include other potential causes.

## Conclusions

Not all ST-segment elevations are indicative of myocardial infarction (MI). In patients with Brugada pattern type ST elevation, electrolyte abnormalities such as hyperkalemia should be considered, as correction of these abnormalities results in the normalization of EKG changes.
